# Polytherapeutic strategies with oncolytic virus–bortezomib and adjuvant NK cells in cancer treatment

**DOI:** 10.1098/rsif.2020.0669

**Published:** 2021-01-06

**Authors:** Angelica P. Aspirin, Aurelio A. de los Reyes V, Yangjin Kim

**Affiliations:** 1 Institute of Mathematics, University of the Philippines Diliman, C.P. Garcia St., U.P. Campus, Diliman, 1101 Quezon City, Philippines; 2 Department of Mathematics, Konkuk University, 120 Neungdong-ro, Gwangjin-gu, Seoul 05029, Republic of Korea; 3 Mathematical Biosciences Institute, Columbus, OH, USA

**Keywords:** cancer model, optimal control theory, bortezomib, NK cells, oncolytic virus

## Abstract

Proteasome inhibition and oncolytic virotherapy are two emerging targeted cancer therapies. Bortezomib, a proteasome inhibitor, disrupts the degradation of proteins in the cell leading to accumulation of unfolded proteins inducing apoptosis. On the other hand, oncolytic virotherapy uses genetically modified oncolytic viruses (OV) to infect cancer cells, induce cell lysis, and activate an antitumour response. In this work, optimal control theory is used to minimize the cancer cell population by identifying strategic infusion protocols of bortezomib, OV and natural killer (NK) cells. Three different therapeutic protocols are explored: (i) periodic bortezomib and single administrations of both OV and NK cells therapy; (ii) alternating sequential combination therapy; and (iii) NK cell depletion and infusion therapy. In the first treatment scheme, early OV administration followed by well-timed adjuvant NK cell infusion maximizes antitumour efficacy. The second strategy supports timely OV infusion. The last treatment scheme indicates that transient NK cell depletion followed by appropriate NK cell adjuvant therapy yields the maximal benefits. Relative doses and administrative costs of the three anticancer agents for each approach are qualitatively presented. This study provides potential polytherapeutic strategies in cancer treatment.

## 1. Introduction

Cancer is a group of diseases characterized by abnormal cell growth. Toxicity and resistance are common occurrences in conventional therapeutic approaches to cancer such as chemotherapy and radiation therapy due to the poor distinction between cancer cells and normal cells, consequently damaging healthy cells [1–3], and thus emphasizing a need to develop novel and more effective strategies to treat cancer. Targeted cancer therapy, a recent development in the study of cancer, works by attacking the intracellular mechanisms such as signalling pathways that enable cancer cell proliferation and survival [4,5]. As a result, healthy normal cells are left unharmed from the toxicity of the treatment, making it safer and more efficient. Due to its target-specific nature, this novel strategy is growing to be a promising choice for cancer treatment, usually in combination with other standard cancer therapeutics [6].

Cancer cells require increased protein synthesis and degradation for aggressive growth [7,8]. The ubiquitin–proteasome system facilitates the degradation of most of the proteins and thus plays a vital role in maintaining cellular function and homeostasis [8,9]. The implication of the ubiquitin–proteasome system in protein degradation puts focus on proteasome inhibition as an approach to treat cancer [7,8]. Since timed degradation and recycling of proteins is critical for cell viability [10], proteasome inhibitors aim to disrupt this process, resulting to the accumulation of ubiquitin-tagged proteins and the induction of endoplasmic reticulum stress [11] in cancer cells, ultimately leading to *apoptosis*. The first-in-class proteasome inhibitor used for clinical use is *bortezomib*—a peptide-based, reversible proteasome inhibitor and a Food and Drug Administration (FDA)-approved drug for multiple myeloma and lymphoma. The efficacy of bortezomib as a single agent in cancer treatment is limited. In particular, bortezomib does not consistently induce apoptosis in melanoma cells and occasionally even upregulates anti-apoptotic factor [12]. However, its efficacy increases when combined with other therapeutic agents [9,13,14].

Another emerging targeted cancer treatment is oncolytic virotherapy, which uses replication-competent viruses to destroy cancer cells [3,15]. Oncolytic viruses (OVs) are genetically modified to specifically infect and replicate in cancer cells while causing minimal damage to healthy normal cells [16,17]. When OVs infect and destroy cancer cells, they also evoke adaptive antitumour responses from the immune system [18]. Several species of OVs are now under multiple clinical trials to test their efficacy and safety [2,8]. For example, T-Vec, a modified *herpes simplex virus 1* (oHSV), gained FDA approval for treatment of advanced melanoma patients in 2015 [8,18,19]. Oncolytic virotherapy alone, however, offers limited antitumour efficacy due to early virus clearance from OV-induced immune response [4]. To address this matter, combination treatments involving OVs and several established chemotherapeutic drugs are being investigated for their synergistic effects to tumour cell killing [18,20].

In 2014, a study by Yoo *et al.* [8] on the oHSV–bortezomib combination treatment for different types of solid cancer showed that bortezomib induction of unfolded protein response in tumour cells promoted nuclear localization of the virus *in vitro*, increasing viral replication and synergistic tumour cell killing [8,21]. In 2016, a follow-up study [22] demonstrated that the combination treatment for glioblastoma induced *necroptosis*—a programmed form of inflammatory cell death (necrosis) stimulated by the secretion of cytokines resulting in inflammation. The increase in pro-inflammatory cytokine secretion also activated an antitumour immune response from *natural killer* (NK) cells which sensitized the tumour cells to NK-mediated apoptotic death and promoted overall therapeutic efficacy [21,22]. NK cells, a type of lymphocyte and a component of the innate immune system, are essential in host immunity against cancer [23]. These cells have the ability to recognize cancer even without the presence of tumour-specific antigens, which makes them effective for cancer treatment. Their potential in immune surveillance and immunotherapy has encouraged various studies in NK cell activity to understand and exploit their functions for cancer treatment, infections and other pathologic conditions [24–29].

From the results in [8,22], Kim *et al.* [21] expressed the dynamics of cancer cells under OV–bortezomib treatment using a mathematical model. The paper considered the role of NK cells in the overall antitumour efficacy of the OV–bortezomib combination treatment. Kim *et al.* [30] extended the model to include the intracellular mechanisms that govern the signalling pathways of the cancer cells under the treatment. In both papers, the treatment protocols greatly affect the growth of the cancer cell population and thus can also dictate the level of success of the treatment. Our modelling framework uses the concept of *optimal control theory*, a mathematical tool that deals with complex biological systems that can be controlled by an external agent [31]. Mathematical modelling and optimal control theory are often used hand-in-hand in epidemiology and cancer research to construct effective treatment protocols against various types of diseases. The papers in [32–34] are some of the earlier works that involved the application of optimal control in cancer treatment. These studies modelled the growth of the tumour under chemotherapy and formulated an optimal control problem that minimized the tumour cell population and drug dosage. Recent applications of optimal control theory in cancer research can be found in [35,36], where the approach is utilized to maintain upregulated levels of miR-451 to prevent cell migration in glioblastoma. In the current study, the goal is to identify appropriate treatment schedules and doses for bortezomib, OV and exogenous NK cells so that the antitumour efficacy is maximized while treatment cost and systemic toxicity are minimized. Three different therapeutic protocols are explored: (i) periodic bortezomib and single administrations of both OV and NK cells therapy; (ii) alternating sequential combination therapy; and (iii) NK cell depletion and infusion therapy. An optimal control problem is formulated for each strategy in order to minimize cancer cell population and total administration cost of the three anticancer agents. Simulation results provide effective treatment schedule and drug dosage taking into account antitumour efficacy for each scheme.

In the subsequent section, we develop a modified mathematical model of the combination (bortezomib–OV–NK) cancer therapy based on Kim *et al*. [21] for optimal treatment strategies. An optimal control problem is formulated so that the cancer cell population is minimized with the least possible administration costs for the proposed treatments. In the results and discussion section, different strategies are explored and the optimal solutions in terms of the cancer cell population at the end of the treatment period, doses and relative cost of bortezomib, OV and exogenous NK cells infusions are discussed. The last section summarizes the findings and presents future research directions.

## 2. Material and methods

### 2.1. Mathematical model

**Figure 1.**
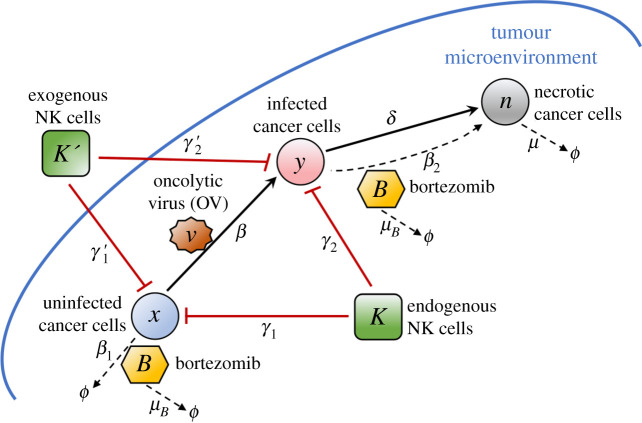
A regulatory network in the tumour microenvironment with bortezomib-assisted OV treatment and NK cell intervention. Arrows (black) indicate activation and induction while hammerheads (red) indicate inhibition.

In this study, the interactions between cancer cells and treatment using bortezomib (*B*), oncolytic virus (*v*) and NK cells (*K*) are explored using a mathematical model. Three types of cancer cells are considered: *uninfected* (*x*), *virus-infected* (*y*) and *necrotic* (*n*) cells. The regulatory network in figure 1 illustrates the dynamics of the interacting components. OVs initially infect and replicate within cancer cells. After rapid viral replication, the infected cancer cells rupture and release new viruses which infect other cancer cells. Ruptured cancer cells become necrotic cancer cells and activate endogenous NK cells (*K*) as an antitumour response. The cytokines IFN-*γ* and TNF-*α* from necrotic cancer cells are responsible for the activation of NK cells [22]. Bortezomib, on the other hand, induces apoptosis in uninfected cancer cells via proteasome inhibition. Meanwhile, the combination of bortezomib and OV induces necroptosis in infected cancer cells, which also activates endogenous NK cells as an antitumour response. Viral replication within infected cancer cells is enhanced in the presence of bortezomib and also improves the activation of endogenous NK cells. Finally, exogenous NK cells (*K*′) are injected into the tumour to aid in killing uninfected and infected cancer cells.

The time evolution of different types of cancer cells (uninfected, infected and necrotic cells) considers proliferation, virus infection and clearance process. The proliferation rate of uninfected cells is *λ* and the carrying capacity is *x*_0_. The following rates are significant model parameters: *β* is the infection rate, *δ* is the infected cell lysis rate, *μ* is the removal rate of dead cells, *β*_1_ is the bortezomib-induced apoptosis of tumour cells, *β*_2_ is the bortezomib-induced necroptotic cell death rate of infected cells, *γ*_1_ and *γ*_2_ are the killing rates of uninfected and infected cells by endogenous NK cells, respectively, and *γ*_1_′ and *γ*_2_′ are the killing rates of uninfected and infected cells by exogenous NK cells, respectively. The combination of OV and bortezomib induces necrotic cancer cells to recruit NK cells facilitated by cytokines IFN-*γ* and TNF-*α* from necrotic cells [22]. The recruitment rate is assumed to be proportional to $n(B/(k_{B}+B))$, where *k*_*B*_ is constant [21]. NK cells are injected into the tumour as adjuvant therapy at a rate of *u*_*K*_′ at specific period [*t*_*K*′_, *t*_*K*′_ + *τ*]. The virus is considered to be replication-competent injected at a rate *u*_*V*_ at a particular time [*t*_*v*_, *t*_*v*_ + *τ*], and *b* denotes the number of viral particles released when an OV infected cell dies by lysis. In addition, the bortezomib improves viral replication by a factor proportional to *B*. Bortezomib is supplied at a rate of *u*_*B*_ with its consumption from internalization in both uninfected and infected tumour cells at rates *μ*_1_ and *μ*_2_, respectively, and natural decay at a rate of *μ*_*B*_ [21]. The dynamics of the network can be described by a system of coupled ordinary differential equations as follows:

2.1 $$\left.\begin{array}{rl} & \;\frac{\text{d}x}{\text{d}t}=\lambda x\left(1-\frac{x}{x_{0}}\right)-\beta xv-\beta_{1}xB-\gamma_{1}xK-\gamma_{1}^{\prime }xK^{\prime },\\ & \;\frac{\text{d}y}{\text{d}t}=\beta xv-\delta y-\beta_{2}yB-\gamma_{2}yK-\gamma_{2}^{\prime }yK^{\prime },\\ & \;\frac{\text{d}n}{\text{d}t}=\delta y+\beta_{2}yB-\mu n,\\ & \;\frac{\text{d}K}{\text{d}t}=\lambda_{1}n\left(1+\alpha_{2}\frac{B}{k_{B}+B}\right)-\mu_{K}K,\\ & \;\frac{\text{d}K^{\prime }}{\text{d}t}=u_{K^{\prime }}I_{\left[t_{K\prime },t_{K^{\prime }}+\tau \right]}-\mu_{K^{\prime }}K^{\prime },\\ & \;\frac{\text{d}v}{\text{d}t}=u_{V}I_{\left[t_{v},t_{v}+\tau \right]}+b\delta y(1+\alpha_{1}B)-\gamma v\\ \;\text{and}\; & \;\frac{dB}{dt}=u_{B}-(\mu_{1}x+\mu_{2}y)\frac{B}{k_{B}+B}-\mu_{B}B. \end{array}\right\}$$

Table 1 provides the full list of parameters, their meaning and corresponding values used in the model.

**Table 1.** Parameters of the non-dimensionalized model.

parameter	description	value	reference
*λ*	proliferation rate of tumour cells	1.8 × 10^−1^	[37]
*x*_0_	carrying capacity of uninfected tumour cells	9.98 × 10^−1^	[21]
*β*	virus infection rate	2.332 × 10^−3^	[37]
*δ*	infected cell lysis rate	2 × 10^−1^	estimated
*b*	burst size of infected cells	20	estimated
*α*_1_	bortezomib-induced viral replication rate	1.0	[21]
*λ*_1_	endogenous NK cell activation rate	1.1 × 10^−3^	[21]
*α*_2_	by bortezomib	2.0	[21]
*γ*	clearance rate of viruses	1.8 × 10^−3^	[37]
*μ*	removal rate of dead cells	1.04 × 10^−1^	[37]
*β*_1_	bortezomib-induced apoptosis rate	1.5 × 10^−2^	estimated
*β*_2_	bortezomib-induced necroptosis rate	3 × 10^−2^	estimated
	killing rate of uninfected tumour cells		
*γ*_1_	by endogenous NK cells	1 × 10^−2^	[21]
*γ*′_1_	by exogenous NK cells	1.3 × 10^−2^	[21]
	killing rate of infected tumour cells		
*γ*_2_	by endogenous NK cells	2.9	[21]
*γ*′_2_	by exogenous NK cells	1.3 × 10^−1^	[21]
	consumption rate of bortezomib		
*μ*_1_	by uninfected tumour cells	2.075 × 10^−1^	[21]
*μ*_2_	by infected tumour cells	2.075 × 10^−1^	[21]
*k*_*B*_	Hill-type parameter	1.0	[21]
*μ*_*B*_	decay rate of bortezomib	1.5 × 10^−1^	estimated
*μ*_*K*_	death rate of endogenous NK cells	4.1 × 10^−3^	[38,39]
*μ*_*K*′_	death rate of exogenous NK cells	4.1 × 10^−3^	[38,39]

### 2.2. Optimal control formulation

In this work, the modelling framework uses *optimal control theory* to identify strategic infusion protocols for bortezomib, OV and exogenous NK cells that will control the proliferation of cancer cells. Specifically, the goal is to find appropriate infusion rates *u*_*B*_, *u*_*V*_ and *u*_*K*′_ (referred to as *controls*) such that the cancer cell population is minimized while treatment administration cost is also at a minimum. The cost of treatment administration is represented as a linear combination of $u_{B}^{2}$, $u_{V}^{2}$ and $u_{K^{\prime }}^{2}$. The optimal control problem is formulated as a minimization problem, i.e. minimize the objective functional *J* such that

2.2 $$\begin{array}{rl} & J(u_{B}(t),u_{V}(t),u_{K^{\prime }}(t))=\\ & \;\int_{t_{0}}^{t_{\;f}}\left[x(t)+y(t)+\frac{C_{B}}{2}u_{B}^{2}(t)\chi_{B}(t)\;+\frac{C_{V}}{2}u_{V}^{2}(t)\chi_{V}(t)+\frac{C_{K^{\prime }}}{2}u_{K^{\prime }}^{2}(t)\chi_{K^{\prime }}(t)\right]\text{d}t, \end{array}$$

subject to system (2.1). The weight parameters *C*_*B*_, *C*_*V*_ and *C*_*K*′_ are the measures of the costs of implementing the respective treatments relative to minimizing the cancer cell population *x*(*t*) + *y*(*t*). In addition, *χ*_*B*_(*t*), *χ*_*V*_(*t*) and *χ*_*K*′_(*t*) are indicator functions for bortezomib, OV and exogenous NK cell administration, respectively. These functions are either one (if administered) or zero (if not administered). Optimal controls $u_{B}^{*}$, $u_{V}^{*}$ and $u_{K^{\prime }}^{*}$ are sought such that the objective functional is minimized, i.e.

$$J(u_{B}^{*}(t),u_{V}^{*}(t),u_{K^{\prime }}^{*}(t))=\underset{\Omega }{\min}\;J(u_{B}(t),u_{V}(t),u_{K^{\prime }}(t)),$$

where

$$\begin{array}{rl} \Omega & =\{u_{B},u_{V},u_{K^{\prime }}\in L^{2}([t_{i},t_{i}+\tau ])\;|\;0\leq u_{i}(t)\leq u_{i}^{\max},\\ & t\in [t_{i},t_{i}+\tau ],\;i=B,V,K^{\prime }\}. \end{array}$$

The values of the controls are bounded by $u_{B}^{\max}$, $u_{V}^{\max}$ and $u_{K^{\prime }}^{\max}$ to impose maximum allowed rates as well as doses per infusion. It is important to note that the existence of optimal controls is guaranteed from the results in control theory [40]. The integrand in (2.2) is convex on Ω with respect to *u*_*B*_, *u*_*V*_ and *u*_*K*′_. Hence, Pontryagin’s maximum principle [41] can be applied to the minimization problem to obtain the necessary conditions for the optimality system. The optimal control problem is numerically solved using an iterative process called *forward-backward sweep method* [31] shown to be convergent [42]. It considers the optimal control problem as a two-point boundary system and uses the fourth-order Runge–Kutta method to solve the optimality system. Given the initial conditions for the state variables and estimates for the controls, the state equations are solved forward in time. The adjoint equations are then solved backward in time using the transversality conditions and the obtained state values. The control values are attained by substituting the new state and adjoint values to the characterization of the optimal controls. Finally, the controls are updated using a convex combination of the previous and current control values. The process continues until the stopping criterion is achieved. Further details on optimal control considered in this study can be found in the electronic supplementary material.

Three different therapeutic protocols are explored to minimize the number of cancer cells and anticancer treatment administration cost. The first scheme considers a periodic infusion of bortezomib coupled with single administration of OV and NK cells. Efficacy of this therapy is investigated by varying administration period for the OV and NK cells. The second strategy examines an alternating sequential therapy in which different combinations of two-round infusions for each anticancer agents are administered within a given treatment period. The third therapy investigates appropriate time period(s) for (exogenous) NK cell administration after (endogenous) NK cell depletion while administering a periodic bortezomib infusion. In this strategy, a new drug *D* that eliminates NK cells is introduced and the change in its concentration is represented by

2.3 $$\frac{dD}{dt}=\lambda_{D}-\mu_{D}D,$$

where *λ*_*D*_ is the infusion rate of the drug while *μ*_*D*_ is its decay rate. The term *γ*_*D*_*DK*, where *γ*_*D*_ represents the killing rate of *D*, is subtracted from the differential equations of *K* and *K*′ to represent the loss of NK cells from the drug, thus,

2.4   dKdt=λ1n1+α2BkB+B−μKK−γDDKand dK′dt=uK′I[tK′,tK′+τ]−μK′K′−γDDK′.

To demonstrate the antitumour capabilities of the combined methods, the optimal control problem considers minimizing the objective functional

2.5 $$J(u_{V}(t),u_{K^{\prime }}(t))=\int_{t_{0}}^{t_{\;f}}\left[x(t)+\frac{C_{V}}{2}u_{V}^{2}(t)\chi_{V}(t)+\frac{C_{K^{\prime }}}{2}u_{K^{\prime }}^{2}(t)\chi_{K^{\prime }}(t)\right]\text{d}t,$$

subject to systems (2.1) and (2.3) with modifications (2.4). In this case, bortezomib is periodically applied but the dose is constant in every infusion, while OV and exogenous (exo-)NK cell doses are determined by optimal control. Details on the control formulation can be found in the electronic supplementary material.

For the aforementioned therapies, the *dose per infusion* of each control (anticancer agent) is computed as the average area under the optimal curves. The *total relative costs* of optimal infusions of bortezomib, OV and exogenous NK cells are represented by

$$\frac{C_{B}}{2}\sum_{i=1}^{N}{\left(u_{B,i}^{*}\right)}^{2}\cdot \text{d}t,\;\frac{C_{V}}{2}{\left(u_{V}^{*}\right)}^{2}\cdot \text{d}t\;\text{and}\;\frac{C_{K^{\prime }}}{2}{\left(u_{K^{\prime }}^{*}\right)}^{2}\cdot \text{d}t,$$

respectively, where d*t* is the timestep and in the case of the bortezomib control, *N* is the total number of infusions over the duration of treatment. The default weight parameters used for the controls are *C*_*B*_ = 10^−3^, *C*_*V*_ = 10^−8^ and *C*_*K*′_ = 10^−3^. Furthermore, the antitumour efficacy (ATE) for each case is computed as follows:

$$\mathrm{ATE}=1-x_{t_{\;f}},$$

where $x_{t_{\;f}}$ is the uninfected cancer cell population at the final time. The ATE plot is normalized by the maximum ATE value among all cases.

## 3. Results and discussion

### 3.1. Periodic bortezomib and single administration of both oncolytic virus and natural killer cells therapy

**Figure 2.**
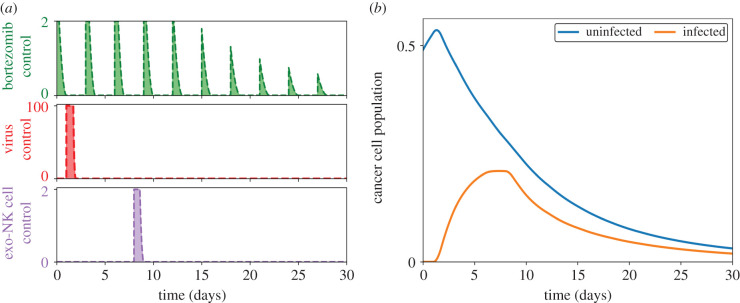
(*a*) Control profiles of bortezomib (top frame, green, dashed), OV (middle frame, red, dashed) and exogenous NK cells (bottom frame, purple, dashed) with the optimal dose for each control infusion depicted as shaded region. Bortezomib is administered every 3 days while OV and NK cells are singly administered on day 1 and day 8, respectively. (*b*) Effect of the controls on the cancer cell population (uninfected, blue; infected, yellow).

Bortezomib infusion is done periodically to minimize toxicity and to avoid unwanted side effects from drug overdose. On the contrary, OV and exogenous NK cells are both administered once, at *t*_*V*_ until *t*_*V*_ + *τ* and *t*_*K*′_ until *t*_*K*′_ + *τ*, respectively. Furthermore, to avoid unprecedented side effects of drug complications, concomitant administrations of anticancer agents are avoided. The profile of the controls and the corresponding effect on the cancer cell population are shown in figure 2. Here, bortezomib is injected every 3 days and we set *t*_*V*_ = 1, *t*_*K*′_ = 8 and *τ* = 1.

The first administration of bortezomib is done at the start of the treatment period. Afterwards, a single dose of OV is injected. At this point, the uninfected cancer cell population starts to decrease due to OV infection. Correspondingly, this translates to an increase in the infected cancer cell population. The next two bortezomib infusions aid in cancer cell killing through apoptosis for uninfected cancer cells and necroptosis for infected cancer cells. The addition of exogenous NK cells halts the increase of the infected cancer cell population and also further declines the uninfected cancer cell population. At the end of treatment, the uninfected and infected cancer cell populations have significantly decreased, successfully demonstrating the ability of the OV–bortezomib combination treatment and additional NK cell infusion in cancer cell killing.

**Figure 3.**
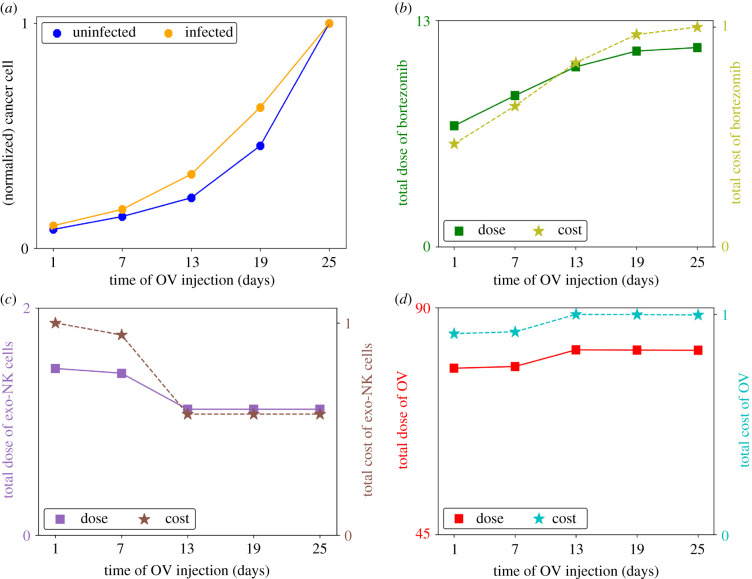
(*a*) Normalized cancer cell population at *t* = 30, and total doses and relative costs of (*b*) bortezomib, (*c*) NK cell and (*d*) OV administrations at different infusion time points of OV control. Delaying OV administration which entails lower exo-NK infusion dose but higher bortezomib and OV doses, reduces antitumour efficacy.

Since OV and exogenous NK cells are administered once, it is important to identify the appropriate time of injection for the anticancer agents so that their antitumour efficacies are maximized. We administered OV and exogenous NK cell treatments at varying time points and examined the effect in cancer cell killing. Figure 3*a* depicts the normalized cancer cell population at the final time and figure 3*b*–*d* shows the doses and relative costs of bortezomib, exogenous NK cells and OV infusions, respectively, corresponding to the different time instances of OV injection *t*_*V*_. For cases *t*_*V*_ = 1 and *t*_*V*_ = 7, the uninfected cancer cell population is still increasing, thus, only low doses of OV are needed for treatment. Correspondingly, the presence of infected cancer cells at the time of NK cell infusion requires higher doses of NK cells to minimize both cancer cell populations. When *t*_*V*_ ≥ 13, the uninfected cancer cell population is relatively high. Therefore, a higher dose of OV is needed. However, in this case, there are no infected cancer cells yet at the time of NK cell infusion (*t*_*K*′_ = 8), therefore, a lower dose of NK cells is chosen. When OV administration is delayed, the needed bortezomib dose increases as a response to the increasing uninfected cancer cell population. Therefore, early OV infusion implies lower bortezomib and OV administration costs but higher NK cell administration cost while delayed OV infusion implies the opposite. The increase in the cancer cell population as shown in figure 3*a* suggests that the antitumour efficacy of OV is reduced when it is administered at a later time in the treatment. The strength of OV-mediated cancer cell killing relies on viral replication inside the cancer cells, therefore delaying its infusion will shorten the allowable time for the antitumour activity of OVs to take effect, resulting in a decreased productivity in cancer cell killing.

**Figure 4.**
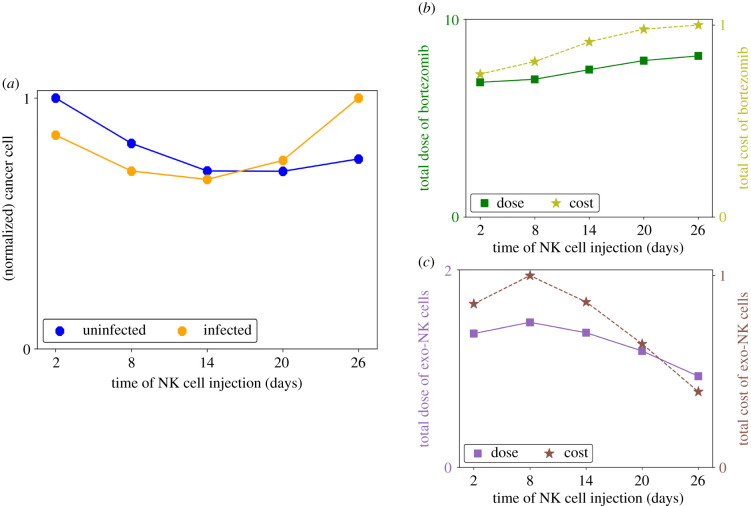
(*a*) Normalized cancer cell population at *t* = 30, and total doses and relative costs of (*b*) bortezomib and (*c*) NK cell administrations at different infusion time points of exogenous NK cells. Early adjuvant exo-NK infusion does not take advantage of OV–bortezomib synergy and delayed administration requires higher bortezomib dose with lower infusion dose of exo-NK cells leading to a poor NK-cell-mediated killing. Intermediate time points for adjuvant exo-NK administration may improve antitumour efficacy.

Figure 4*a* shows the cancer cell population at the final time and figure 4*b*,*c* shows the bortezomib and NK cell doses and relative costs with respect to the varying injection time points of NK cells. Since OV control administration (*t*_*V*_ = 1) always precedes exogenous NK cell infusion, the addition of exogenous NK cells will not affect the optimal OV dose. When *t*_*K*′_ = 2, the uninfected cancer cell population is still high and the infected cancer cell population is relatively low from the recent OV administration. Hence, a high dose of exogenous NK cells is needed to minimize the cancer cell population. Moreover, early elimination of cancer cells from NK cell-mediated killing means that a lower dose of bortezomib is needed for the rest of the treatment. Thus, the relative cost of exogenous NK cell administration is high and the total relative cost of bortezomib administration is low. When *t*_*K*′_ = 8, infected cancer cells have increased due to viral replication and infection, hence a higher dose of exogenous NK cells is needed to minimize the population. When NK cell administration is delayed at the later part of the treatment period, the levels of both cancer cells are already low due to the initial OV–bortezomib infusions. Hence, adjuvant infusion at this time only requires lower doses of exogenous NK cells to minimize the cancer cell population. Conversely, a higher dose of bortezomib is needed so that the increased infected cancer cell population is minimized before exogenous NK cells are administered. Therefore, delayed NK cell infusion implies lower NK cell administration cost but higher bortezomib administration cost. The cancer cell population in figure 4*a* exhibits a non-monotonic behaviour with respect to the different infusion time points of NK cells. For early adjuvant infusion (*t*_*K*′_ = 2, 8), the high dose of NK cells prematurely eliminates the infected cancer cells and does not take advantage of the antitumour efficacy of the OV–bortezomib combination treatment, thus leading to poor outcome. On the other hand, by delaying NK cell administration (*t*_*K*′_ = 26), the low dose of NK cells weakens the efficacy of the NK cell-mediated killing, also leading to poor results. Therefore, intermediate time points of NK cell administration are more effective in cancer cell killing. In this case, the antitumour efficacy of the OV–bortezomib treatment is maximized while the additional higher dosage of NK cells is enough to effectively eliminate the remaining cancer cells.

### 3.2. Alternating sequential combination therapy

**Figure 5.**
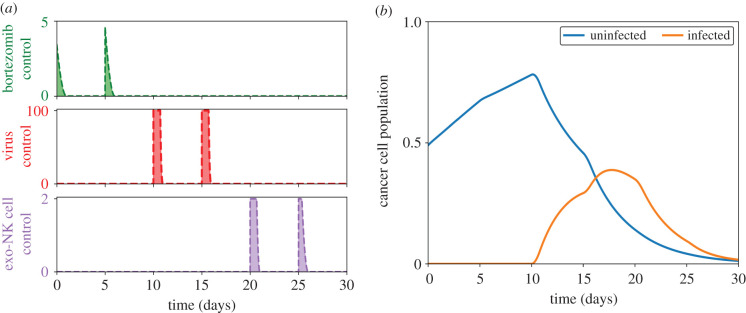
(*a*) Optimal treatment scheme **BBVVKK** where bortezomib is administered at day 0 and 5, OV is infused at day 10 and 15, and NK cells therapy is performed at day 20 and 25. (*b*) Time evolution of uninfected and infected cells under **BBVVKK** therapy.

The optimal control scheme in the previous discussion shows decreasing doses of bortezomib for each succeeding infusion. In fact, the doses of bortezomib become too small at the latter part of the treatment period. Realistically, having to come back regularly in the clinic and be injected by only small doses of the treatment can be painful, inconvenient, and not cost-effective for both patients and clinicians. In this section, instead of periodic infusions of bortezomib and single administrations of OV and NK cells, we assume two rounds of infusions for each anticancer agent and distribute them for the entire duration of treatment at equally spaced periods. In particular, each treatment is administered at day 0, 5, 10, 15, 20 and 25, respectively. For example, consider two initial injections of bortezomib (BB), followed by two administrations of OV (VV) and two infusions of NK cells (KK), then this scheme will be labelled as **BBVVKK** (refer to figure 5). There are 12 different combinations of treatment considered and investigated to assess which scheme is the most effective and cost-efficient in cancer cell killing.

**Figure 6.**
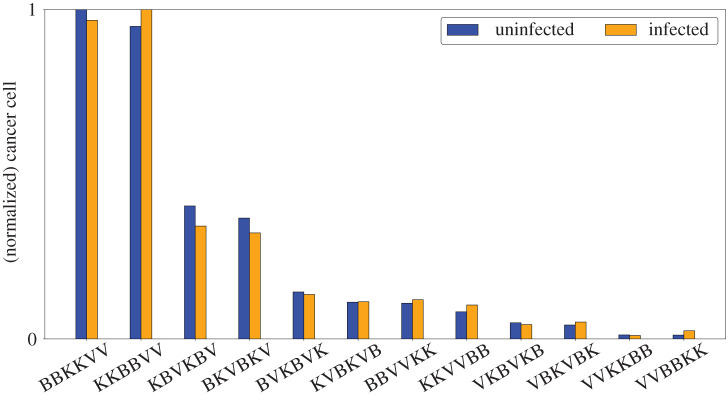
Normalized cancer cell population at *t* = 30 corresponding to different alternating schemes of bortezomib, OV and NK cells. Delayed administration of OV (**BBKKVV** and **KKBBVV**) results in poor cancer cell killing while early administration (**VVKKBB** and **VVBBKK**) enhances antitumour efficacy.

**Figure 7.**
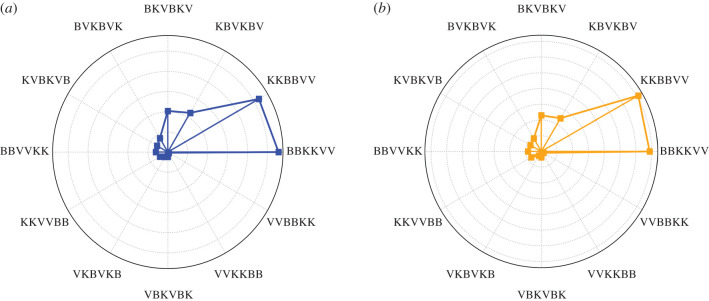
Polar representation of the normalized (*a*) uninfected and (*b*) infected cancer cell population at *t* = 30 corresponding to the different alternating schemes of bortezomib, OV and NK cells. Delayed administration of OV (**BBKKVV** and **KKBBVV**) yields poor anticancer efficacy.

**Figure 8.**
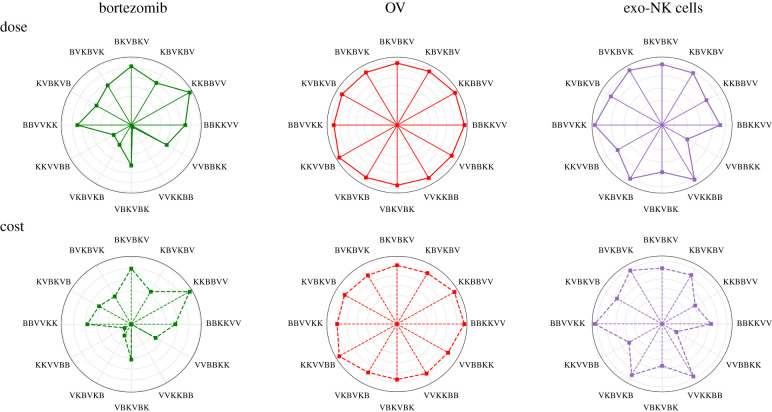
Polar representation of the doses (top) and relative costs (bottom) of bortezomib, OV and exogenous NK cells corresponding to the different alternating treatment schemes. Higher dose of bortezomib is needed when administered before OV treatment and large dose of exo-NK is required when OV infusion precedes exo-NK infusion.

The normalized cancer cell populations corresponding to the different schemes are shown in figure 6. It suggests that combinations **BBKKVV** and **KKBBVV** are the least effective in cancer cell killing. On the other hand, the combination **VVBBKK** is the most effective scheme in eliminating uninfected cancer cells while **VVKKBB** is most effective at killing infected cancer cells. An alternative visualization of the results in this strategy is depicted in figures 7 and 8. In the figures, the centre of the circle represents the 0 value and the lengths of the lines connecting the centre and the points represent the numerical values of the attribute corresponding to each scheme. Thus, shorter lines represent smaller values, while longer lines represent larger values. In figure 7, it can be observed that the cancer cell population is higher when OV is administered late into the treatment period, e.g. **BBKKVV, KKBBVV, KBVKBV, BKVBKV**. These schemes yield poor anticancer efficacy since they do not take advantage of the antitumour activity from the OVs. By contrast, cancer cell killing is highly productive when OV is administered at the start of treatment, e.g. **VVBBKK, VVKKBB**. These treatment configurations allow ample time for the OVs to infect cancer cells, which also amplifies the efficacy of bortezomib and exogenous NK cells. It is also interesting to note that alternating treatments produce mediocre results in cancer cell killing. Therefore, consecutive infusions of the same treatment is preferred.

The doses of bortezomib, OV and exogenous NK cells and the corresponding costs for the different schemes are shown in figure 8. There is little difference between the doses of OV among all the treatment combinations, which also implies minimal variation in terms of the OV administration cost. Large doses of bortezomib are needed when it is applied before OV, as seen in combinations **KKBBVV**, **BKVBKV**, **BBKKVV**, **BBVVKK**. Without OVs, only uninfected cancer cells are present in the tumour. Therefore, high doses of bortezomib are needed in every infusion to minimize the increasing cancer cell population in the aforementioned treatment combinations. On the other hand, initial infusions of OV and exogenous NK cells in schemes **VVKKBB**, **KKVVBB**, **VKBVKB** already eliminate most of the cancer cells, thus only requiring smaller doses of bortezomib infusions. Therefore, lower bortezomib doses are needed when it is applied at the end of treatment. Exogenous NK cell doses are higher when OV infusion precedes exogenous NK cell injection, e.g. **BBVVKK**, **BVKBVK**, **VVKKBB**, **VKBVKB**. The needed dose of NK cells to eliminate cancer cells increases in the presence of both uninfected and infected cancer cells. The scheme **VVBBKK** requires the least dose of exogenous NK cells. This is expected since the previous infusions of OV and bortezomib have already eliminated most of the cancer cells through necroptosis, thereby requiring only a low dose of exogenous NK cells. As discussed previously, **VVBBKK** and **VVKKBB** are the most effective treatment combinations in cancer cell killing. While both schemes require relatively the same doses of OV, the former requires a higher dose of bortezomib but a lower dose of exogenous NK cells while the latter requires a lower dose of bortezomib but a higher dose of exogenous NK cells.

### 3.3. Natural killer cell depletion and infusion therapy

Finally, the role of NK cells in the overall efficacy of the cancer drug treatments is considered. The capability of NK cells to eliminate cancer cells has already been established in previous studies. However, the NK cell-mediated immune response in infected cancer cells dampens the production of newly produced viruses and thus reduces the OV population. This lessens viral infection and cell lysis, ultimately reducing the efficacy of OV treatment.

**Figure 9.**
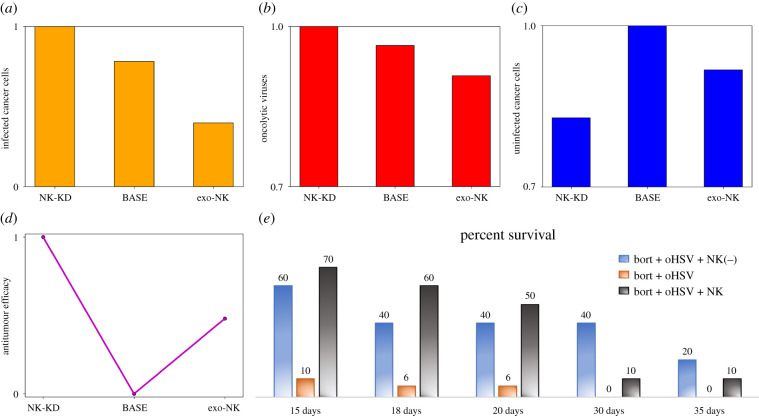
Comparisons of the (*a*) infected cancer cell population, (*b*) OV population and (*c*) uninfected cancer cell population at *t* = 30 of the NK-KD case, BASE case and exogenous NK case. (*d*) Nonlinear behaviour of the overall antitumour efficacy with respect to the three cases. (*e*) Experimental results [21]: survival rate of mice treated with three cases based on Kaplan-Meier survival curves: (i) NK-KD (bortezomib + oHSV + NK(−)), (ii) base (bortezomib + oHSV) and (iii) exo-NK (bortezomib + oHSV + NK).

To test the effect of NK cell knockdown (NK-KD), we introduce a drug *D* for elimination of NK cells in the tumour microenvironment for the entire duration of treatment. As the concentration of *D* increases and saturates, the endogenous NK cell population decreases and is kept close to zero. See equations (2.3)–(2.4). In figure 9, we compare three scenarios involving varying quantities of NK cells in the tumour environment: (i) the NK-KD case represents the absence of both endogenous and exogenous NK cells in the system, (ii) the BASE case represents the system with only endogenous NK cells and (iii) the exogenous NK case indicates the presence of both types of NK cells. The bars in figure 9*a*–*c* show the infected cancer cell, OV and uninfected cancer cell population at the final time. Since NK cells also kill infected cancer cells, their absence allows more infected cancer cells to remain in the tumour environment, as shown in the NK-KD case. This also enhances the activity of the OVs, which leads to increased OV population. By contrast, more NK cells in the tumour promotes stronger NK cell-mediated killing, leading to a decreased number of infected cancer cells. As a result, the OV population is also lower. Interestingly, this observation does not necessarily hold for the uninfected cancer cells. While increased NK cells (exogenous NK case) indeed eliminate more uninfected cancer cells compared to the base case, it is found that eliminating the NK cell-mediated immune response (NK-KD case) from the tumour provides the best results in antitumour efficacy. This is because the absence of NK cells in the tumour environment relieves the infected cancers from the immune cell-mediated attack, enhancing antitumour OV activity which leads to improved cancer cell killing. Therefore, a nonlinear relationship between the level of NK cells in the tumour environment and the overall antitumour efficacy exists, as shown in figure 9*d*. Low levels of NK cells, as in the NK-KD case, promote better antitumour efficacy from increased OV activity. On the other hand, strengthened NK cell-mediated killing due to high level of NK cells, as in the exogenous NK case, effectively eliminates cancer cells. These nonlinear responses and computational results are in good agreement with experimental data (figure 9*e*) [21].

**Figure 10.**
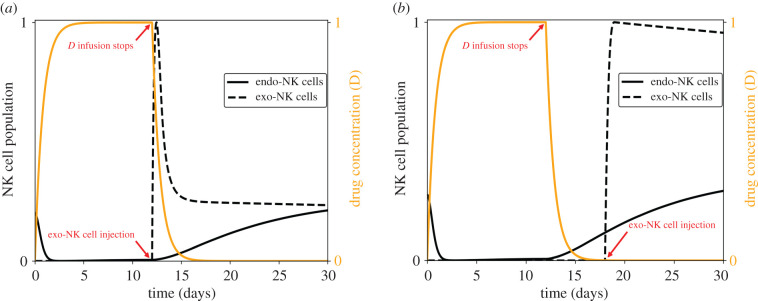
Dynamics of (*a*) immediate exo-NK infusion after NK cell depletion with *t*_1_ = *t*_2_ and (*b*) administration of exo-NK some time after NK cell depletion with *t*_1_ < *t*_2_. Here, *t*_1_ is the time duration for NK cell depletion while *t*_2_ is the infusion time for exo-NK cells.

The nonlinearity of tumour growth with respect to the level of NK cells, and accordingly the strength of NK cell killing, shows that either removing or amplifying the immune cell-mediated attack in the tumour environment leads to better antitumour efficacy. Let *t*_1_ be the final time at which drug *D* infusion stops and *t*_2_ be the time at which exo-NK cell injection starts, i.e. drug *D* is applied from *t* = 0 until *t* = *t*_1_ while optimal exo-NK cell injection is done at *t* = *t*_2_ until *t* = *t*_2_ + 1. Consider two cases: (i) *t*_1_ = *t*_2_ which implies that exo-NK cell injection immediately follows after endogenous (endo-)NK cell depletion and (ii) *t*_1_ < *t*_2_ which implies a time lag between endo-NK cell depletion and exo-NK cell injection. Figure 10 depicts the dynamics of the NK cells for the two cases.

**Figure 11.**
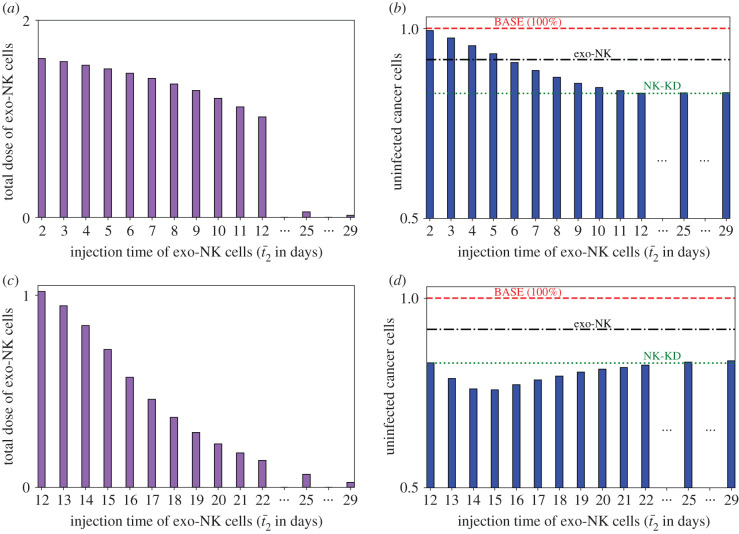
(*a*) Total dose of exo-NK cells obtained from optimal control and (*b*) corresponding levels of uninfected cancer cells at *t* = 30 when exo-NK infusion follows immediately after endo-NK depletion, case 1: ${\bar{t}}_{1}={\bar{t}}_{2}$. (*c*) Total dose of exo-NK cells obtained from optimal control and (*b*) corresponding levels of uninfected cancer cells at *t* = 30 when exo-NK administration is delayed for *t* days after endo-NK depletion for 12 days, case 2: $12={\bar{t}}_{1}<{\bar{t}}_{2}$. Both levels of uninfected cancer cells are relative to the BASE case. The levels of uninfected cancer cells for the BASE, exogenous NK and NK-KD cases are depicted by the red, black and green dashed lines, respectively.

The optimal control scheme is implemented for different values of *t*_2_ and the exo-NK cell doses and uninfected cancer cell population at the final time are compared. The combination strategy takes advantage of both the enhanced antitumour OV activity from the initial depletion of endo-NK cells and the augmented NK cell-mediated tumour killing from the adjuvant infusion. Figure 11*a* illustrates the total dose of exo-NK cells obtained from the optimal control when infusion of exo-NK cells immediately follows after the depletion of endo-NK cells (case 1: ${\bar{t}}_{1}={\bar{t}}_{2}$). Figure 11*b* depicts the scenario when administration of exo-NK cells is delayed for *t* days after endo-NK cell depletion for 12 days (case 2: $12={\bar{t}}_{1}<{\bar{t}}_{2}$). The relative population of the uninfected cancer cells with respect to the BASE case (red, dashed) using the new combination strategy for both cases are depicted in figure 11*c*,*d*, respectively. When the switch from NK cell depletion to NK cell injection occurs early in the treatment (${\bar{t}}_{2}\leq 5$), the relative uninfected cancer population is lower than the BASE case but higher than the exogenous NK case. The intervention of NK cell injection to the depletion process of NK cells occurs too early in these transition time points, thus not taking advantage of the OV-mediated cancer cell killing, leading to worse results than the exogenous NK case. For intermediate transition time points ($6\leq {\bar{t}}_{2}<11$), the uninfected cancer cell population is lower than the exogenous NK case but higher than the NK-KD case. The OV antitumour activity is prolonged in this case, leading to better cancer cell killing efficacy than the exogenous NK case. For all possible combinations of ${\bar{t}}_{1}$ and ${\bar{t}}_{2}$, the uninfected cancer cell population is never lower than the NK-KD case. Note that once the infusion of *D* stops, its concentration starts to decrease. However, when exogenous NK cells are injected immediately after NK cell depletion, a substantial amount of *D* still remains in the system, thus killing the newly injected NK cells. Therefore, immediate NK cell injection provides little to no effect in further decreasing the uninfected cancer cell population. The described behaviour can be seen in figure 11*c*. For case 2, all combinations of ${\bar{t}}_{1}$ and ${\bar{t}}_{2}$ result in lower uninfected cancer cell population compared to the BASE and exo-NK case (figure 11*d*). At $13<{\bar{t}}_{2}<21$, the uninfected cancer cell population resulting from the combination strategy is lower than the NK-KD case. Delaying the exo-NK cell injection allows the concentration of *D* to decrease and the endo-NK cell population to slowly increase. At $13\leq {\bar{t}}_{2}\leq 15$, lower levels of *D* result in better NK cell retention in the tumour environment, leading to increased efficacy of NK cell-mediated killing. At ${\bar{t}}_{2}=15$, the concentration of *D* is almost zero and the higher total NK cell population eliminates the remaining cancer cells, thus achieving the optimal outcome. When ${\bar{t}}_{2}\geq 15$, the uninfected cancer cell population increases again. At these transition time points, the concentration of *D* is already zero and the endo-NK cell population has increased further. The efficacy of the endo-NK cell depletion has reduced and the exo-NK cells are infused too late into the treatment, resulting in a lower antitumour efficacy.

## 4. Conclusion

In this work, we considered the mathematical model developed by Kim *et al.* [21] describing the dynamics of bortezomib, OV and exogenous NK cells in tumour treatment. The focus of this work is to identify different protocols of treatment combinations that effectively use the antitumour capabilities of bortezomib, OV and NK cells using optimal control theory [31,40]. The optimal control problem is formulated such that the cancer cell population and the total administration cost of the three anticancer agents are minimized. Several control strategies are investigated and each antitumour efficacy is examined. The first strategy involved periodic infusions of bortezomib and single administrations of OV and exogenous NK cells. This scheme successfully reduced the cancer cell population. Early infusion of OV led to prolonged antitumour OV activity which resulted to better cancer cell killing. Meanwhile, the strength of NK cell killing followed nonlinear characteristics in relation to the time of its administration. The next control scheme explored alternating sequential infusions of bortezomib, OV and NK cells. Numerical simulations revealed that the OV–bortezomib combination followed by NK cell infusion showed the best result in killing uninfected cancer cells while OV infusion followed by NK cell and bortezomib administrations effectively eliminated infected cancer cells. The third therapy examines the administration of exogenous NK cells after depletion of endogenous NK cells. It has been illustrated that slight delay in NK cell adjuvant infusion after NK cell depletion gave the best results in cancer cell killing. Recent studies showed potential of NK cells in immune surveillance and combined immunotherapy under various pathologic conditions [24–29]. In particular, immunotherapy with NK cells in immunocompromised patients with infectious complications has a great potential with important clinical impact [29].

In this work, we did not take into account the role of chimeric antigen receptor (CAR) in NK cell dynamics, which was shown to be very effective in development of anti-cancer strategies in metastatic cancers. For example, Chen *et al.* showed that careful regional administration of oHSV-1 combined with EGFR-CAR NK cells therapy can be a very effective promising strategy to treat breast cancer brain metastases [43]. In general, OV therapy was shown to be not very effective as a single agent for treatment of cancer and combination therapy can be a better strategy of treating tumours. However, a combination therapy with immune cells such as NK cells may limit viral infection due to host response against infected cells [21,44,45]. It was shown that the combination therapy with CAR NK cells can be effective in targeting heterogeneous tumour populations and cancer stem cells [43], typical causes of relapse, resistance and metastasis in most cancers [46]. Since CAR-modified NK cells in a combination therapy with OVs may disturb the tumour tissue structure, the permeability and replication of OVs can be significantly increased in cancer cells [43]. This may change nonlinear immune response of NK cells in tumour microenvironment. In order to investigate this important aspect of CAR NK cells, we plan to investigate the antitumour efficacy of an OV treatment combined with CAR NK cells and develop optimal strategies of cancer killing in this tumour microenvironment by communicating with experimentalists and assessing the corresponding experimental data. We plan to develop a multi-scale mathematical model that takes into account the detailed multi-scale nature of infectious disease with complex inter- and intra-cellular mechanism and intercorrelation with NK cell therapy.

## Supplementary Material

Supplementary Material for “Polytherapeutic strategies with oncolytic virus-bortezomib and adjuvant NK cells in cancer treatment”

## Data accessibility

This paper has no experimental data and all other data are available within the article.
