# Supplementary material for: Polytherapeutic strategies with oncolytic virus–bortezomib and adjuvant NK cells in cancer treatment
Source: J R Soc Interface. 2021 Jan 6;18(174):20200669. doi: 10.1098/rsif.2020.0669 (PMC7879760; doi:10.1098/rsif.2020.0669)
Supplement: Supplementary Material for “Polytherapeutic strategies with oncolytic virus-bortezomib and adjuvant NK cells in cancer treatment” [file rsif20200669supp1.pdf]

Supplementary Material for "Polytherapeutic strategies with oncolytic virus-bortezomib and adjuvant NK cells in cancer treatment"

Angelica P. Aspirin[1], Aurelio A. de los Reyes V[1], Yangjin Kim [2,3*]

1 Institute of Mathematics, University of the Philippines Diliman, Quezon City, Philippines
2 Department of Mathematics, Konkuk University, Seoul, Republic of Korea
3 Mathematical Biosciences Institute and Department of Mathematics, Ohio State University, Columbus, Ohio, United States of America
*e-mail: ahyouhappy@konkuk.ac.kr

Optimal control problem

Minimizing cancer cells and administration cost of three anticancer agents

In this strategy, the goal is to minimize the cancer cell population and cost that will be incurred in the administration of three anticancer agents, nemely, bortezomib, oncolytic virus and exogenous natural killer cells. This is the governing framework for the two treatment schemes: (1) periodic bortezomib and single administration of both OV and NK cells therapy and (2) Alternating Sequential Combination Therapy. The optimal control problem is then formulated as minimizing the objective functional

$$J\big(u_B(t), u_V(t), u_{K'}(t)\big) = \int_{t_0}^{t_f} \left[x(t) + y(t) + \frac{C_B}{2} u_B^2(t)\chi_B(t) + \frac{C_V}{2} u_V^2(t)\chi_V(t) + \frac{C_{K'}}{2} u_{K'}^2(t)\chi_{K'}(t) \right] dt, \tag{1}$$

over

$$\Omega = \big\{ u_B, u_V, u_{K'} \in \mathscr{L}^2([t_i, t_i+\tau]) \mid 0 \le u_i(t) \le u_i^{\max},\ t \in [t_i, t_i+\tau],\ i = B, V, K' \big\} \tag{2}$$

subject to

$$\begin{aligned}
\frac{dx}{dt} &= \lambda x \left(1 - \frac{x}{x_0}\right) - \beta x v - \beta_1 x B - \gamma_1 x K - \gamma_1' x K', \\
\frac{dy}{dt} &= \beta x v - \delta y - \beta_2 y B - \gamma_2 y K - \gamma_2' y K', \\
\frac{dn}{dt} &= \delta y + \beta_2 y B - \mu n, \\
\frac{dK}{dt} &= \lambda_1 n \left(1 + \alpha_2 \frac{B}{k_B + B}\right) - \mu_K K, \\
\frac{dK'}{dt} &= u_{K'} I_{[t_{K'}, t_{K'}+\tau]} - \mu_{K'} K', \\
\frac{dv}{dt} &= u_V I_{[t_v, t_v+\tau]} + b\delta y (1 + \alpha_1 B) - \gamma v, \\
\frac{dB}{dt} &= u_B - (\mu_1 x + \mu_2 y) \frac{B}{k_B + B} - \mu_B B.
\end{aligned} \tag{3}$$

Note that $\chi_B(t), \chi_V(t)$ and $\chi_{K'}(t)$ are indicator functions for bortezomib, oncolytic virus and exogenous NK cell administration, respectively with values either one (if administered) or zero (if not administered). We have the following theorem:

Theorem 1. *There exist optimal controls $u_B^*(t)$, $u_V^*(t)$, $u_{K'}^*(t)$ and corresponding solutions $x^*(t)$, $y^*(t)$, $n^*(t)$, $K^*(t)$, $K'^*(t)$, $v^*(t)$, $B^*(t)$ that minimize the objective functional* (1) *over* (2)*. Given this optimal solution, there exist adjoint variables $\phi_1, \dots, \phi_7$ satisfying*

$$
\begin{aligned}
\phi_1' &= -1 - \phi_1\left(\lambda - \frac{2\lambda x}{x_0} - \beta v - \beta_1 B - \gamma_1 K - \gamma_1' K'\right) - \phi_2\beta v + \phi_7\left(\frac{\mu_1 B}{k_B + B}\right),\\
\phi_2' &= -1 + \phi_2(\delta + \beta_2 B + \gamma_2 K + \gamma_2 K') - \phi_3(\delta + \beta_2 B) - \phi_6 b\delta(1 + \alpha_1 B),\\
&\quad + \phi_7\left(\frac{\mu_2 B}{k_B + B}\right),\\
\phi_3' &= \phi_3\mu - \phi_4\lambda_1\left(1 + \alpha_2\frac{B}{k_B + B}\right),\\
\phi_4' &= \phi_1\gamma_1 x + \phi_2\gamma_2 y + \phi_4\mu_K,\\
\phi_5 &= \phi_1\gamma_1' x + \phi_2\gamma_2' y + \phi_5\mu_{K'},\\
\phi_6' &= \phi_1\beta x - \phi_2\beta x + \phi_6\gamma,\\
\phi_7' &= \phi_1\beta_1 x + \phi_2\beta_2 y - \phi_3\beta_2 y - \frac{\phi_4\lambda_1 n\alpha_2 k_B}{(k_B + B)^2} - \phi_6 b\delta y\alpha_1\\
&\quad + \phi_7\left((\mu_1 x + \mu_2 y)\frac{k_B}{(k_B + B)^2} + \mu_B\right),
\end{aligned}
$$

with transversality conditions

$$\phi_j(t_i + \tau) = 0 \quad \text{for} \quad j = 1, 2, \dots, 7, \quad i = B, V, K',$$

and optimality conditions

$$
\begin{aligned}
u_B^*(t) &= \min\left(\max\left(0, -\frac{\phi_7}{C_B}\right), u_B^{\max}\right),\\
u_V^*(t) &= \min\left(\max\left(0, -\frac{\phi_6}{C_V}\right), u_V^{\max}\right),\\
u_{K'}^*(t) &= \min\left(\max\left(0, -\frac{\phi_5}{C_{K'}}\right), u_{K'}^{\max}\right).
\end{aligned}
$$

Minimizing cancer cells under adjuvant NK cell infusion therapy

This strategy proposes to minimize the number of uninfected cancer cells and administration cost of OV and NK cells with the assumption or periodic infusion of bortezomib at a constant rate. The optimal control problem minimizes the objective functional

$$J\big(u_V(t), u_{K'}(t)\big) = \int_{t_0}^{t_f}\left[x(t) + \frac{C_V}{2}u_V^2(t)\chi_V(t) + \frac{C_{K'}}{2}u_{K'}^2(t)\chi_{K'}(t)\right]dt, \tag{4}$$

over

$$\Omega_2 = \left\{u_V, u_{K'} \in \mathscr{L}^2\big([t_i, t_i + \tau]\big) \mid 0 \le u_i(t) \le u_i^{\max},\ t \in [t_i, t_i + \tau],\ i = V, K'\right\} \tag{5}$$

subject to

$$
\begin{aligned}
\frac{dx}{dt} &= \lambda x\left(1-\frac{x}{x_0}\right)-\beta xv-\beta_1 xB-\gamma_1 xK-\gamma_1' xK',\\
\frac{dy}{dt} &= \beta xv-\delta y-\beta_2 yB-\gamma_2 yK-\gamma_2' yK',\\
\frac{dn}{dt} &= \delta y+\beta_2 yB-\mu n,\\
\frac{dK}{dt} &= \lambda_1 n\left(1+\alpha_2\frac{B}{k_B+B}\right)-\mu_K K-\gamma_D DK,\\
\frac{dK'}{dt} &= u_{K'}I_{[t_{K'},t_{K'}+\tau]}-\mu_{K'}K'-\gamma_D DK',\\
\frac{dv}{dt} &= u_V I_{[t_v,t_v+\tau]}+b\delta y(1+\alpha_1 B)-\gamma v,\\
\frac{dB}{dt} &= u_B-(\mu_1 x+\mu_2 y)\frac{B}{k_B+B}-\mu_B B,\\
\frac{dD}{dt} &= \lambda_D-\mu_D D.
\end{aligned}
\tag{6}
$$

Theorem 2. *There exist optimal controls $u_V^*(t)$, $u_{K'}^*(t)$ and corresponding solutions $x^*(t)$, $y^*(t)$, $n^*(t)$, $K^*(t)$, $K'^*(t)$, $v^*(t)$, $B^*(t)$, $D^*(t)$ that minimize the objective functional (4) over (5). Given this optimal solution, there exist adjoint variables $\phi_1,\dots,\phi_8$ satisfying such that satisfying*

$$
\begin{aligned}
\phi_1' &= -1-\phi_1\left(\lambda-\frac{2\lambda x}{x_0}-\beta v-\beta_1 B-\gamma_1 K-\gamma_1' K'\right)-\phi_2\beta v+\phi_7\left(\frac{\mu_1 B}{k_B+B}\right),\\
\phi_2' &= \phi_2(\delta+\beta_2 B+\gamma_2 K+\gamma_2 K')-\phi_3(\delta+\beta_2 B)-\phi_6 b\delta(1+\alpha_1 B)+\phi_7\left(\frac{\mu_2 B}{k_B+B}\right),\\
\phi_3' &= \phi_3\mu-\phi_4\lambda_1\left(1+\alpha_2\frac{B}{k_B+B}\right),\\
\phi_4' &= \phi_1\gamma_1 x+\phi_2\gamma_2 y+\phi_4\mu_K+\phi_4\gamma_D D,\\
\phi_5 &= \phi_1\gamma_1' x+\phi_2\gamma_2' y+\phi_5\mu_{K'}+\phi_5\gamma_D D,\\
\phi_6' &= \phi_1\beta x-\phi_2\beta x+\phi_6\gamma,\\
\phi_7' &= \phi_1\beta_1 x+\phi_2\beta_2 y-\phi_3\beta_2 y-\frac{\phi_4\lambda_1 n\alpha_2 k_B}{(k_B+B)^2}-\phi_6 b\delta y\alpha_1\\
&\quad+\phi_7\left((\mu_1 x+\mu_2 y)\frac{k_B}{(k_B+B)^2}+\mu_B\right),\\
\phi_8' &= \phi_4\gamma_D K+\phi_5\gamma_D K'+\phi_8\mu_D,
\end{aligned}
$$

with transversality conditions

$$\phi_j(t_i+\tau)=0 \quad \textit{for} \quad j=1,2,\dots,8,\quad i=V,K',$$

and optimality conditions

$$
\begin{aligned}
u_V^*(t) &= \min\left(\max\left(0,-\frac{\phi_6}{C_V}\right),u_V^{\max}\right),\\
u_{K'}^*(t) &= \min\left(\max\left(0,-\frac{\phi_5}{C_{K'}}\right),u_{K'}^{\max}\right).
\end{aligned}
$$
